# Supplementary material for: Aging Resistance of Biocomposites Crosslinked with Silica and Quercetin
Source: Int J Mol Sci. 2021 Oct 8;22(19):10894. doi: 10.3390/ijms221910894 (PMC8509444; doi:10.3390/ijms221910894)
Supplement: Supplementary file 1 [file ijms-22-10894-s001.zip › ijms-1385033-supplementary.pdf]

International Journal of
Molecular Sciences

Supplementary Material

Aging resistance of biocomposites crosslinked with combination of silica and quercetin

Anna Masek [1]* and Olga Olejnik [1]

[1] Lodz University of Technology, Faculty of Chemistry, Institute of Polymer and Dye Technology, ul. Stefanowskiego 12/16, 90-924 Lodz, Poland
* Correspondence: anna.masek@p.lodz.pl

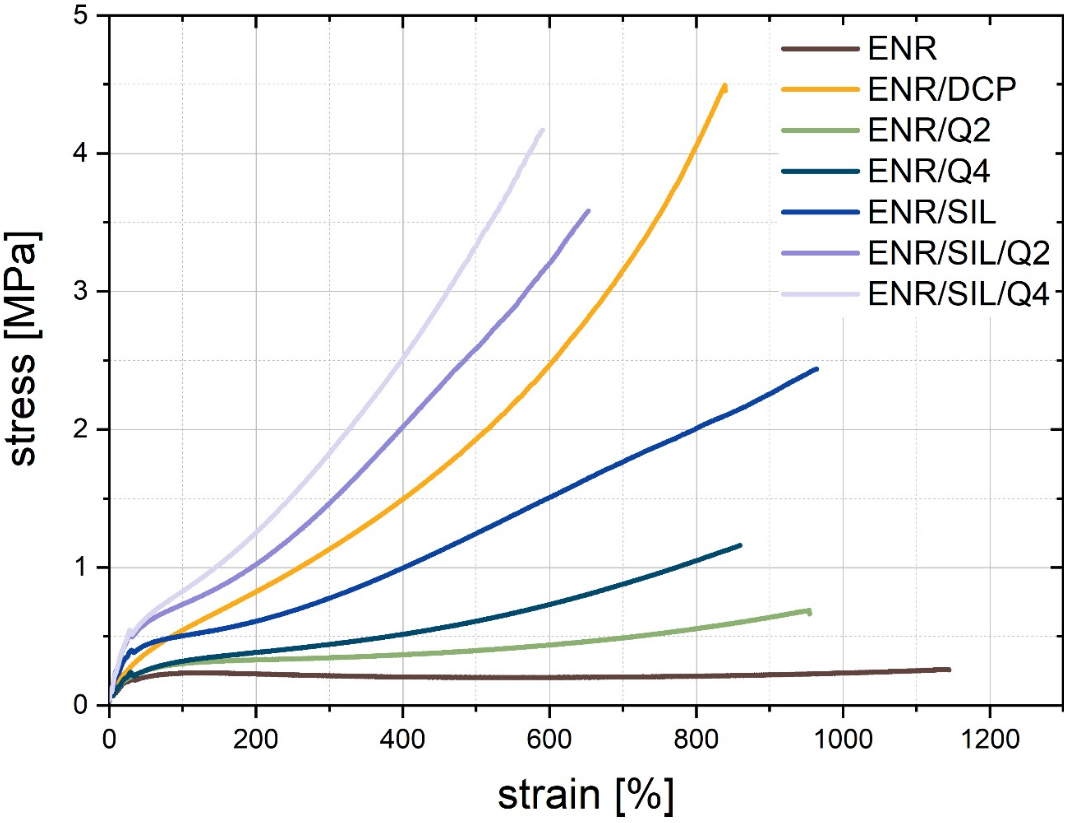

Figure S1. The strain-stress curves of tested ENR-based composites.

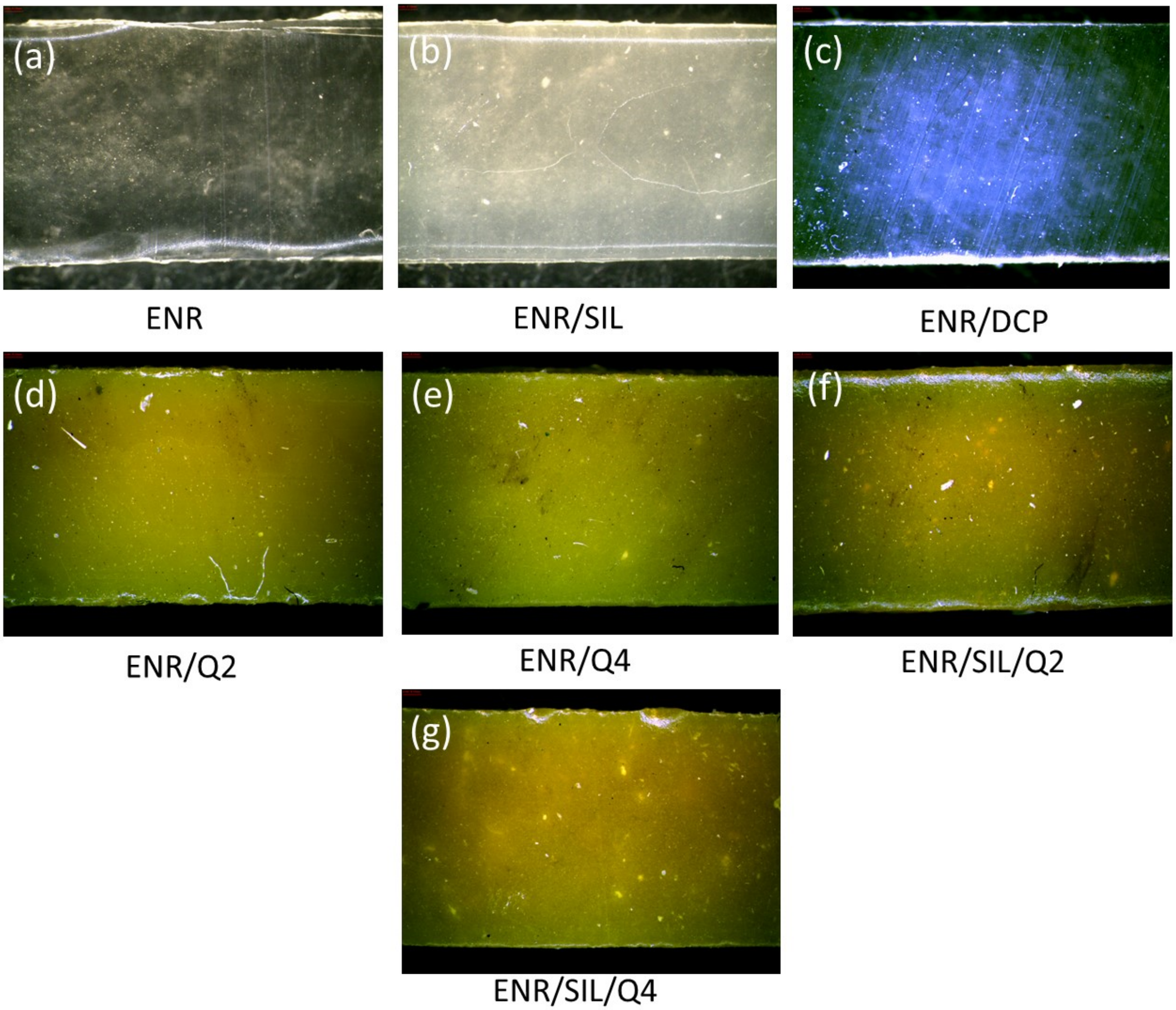

Figure S2. Microscope images of ENR-based composites: (a) pure uncurred ENR, (b) ENR cured with 15 phr of silica, (c) ENR cured with dicumyl peroxide (DCP), (d) ENR cured with 2 phr of quercetin, (e) ENR cured with 4 phr of quercetin, (f) ENR cured with 2 phr of quercetin and 15 phr of silica, (g) ENR cured with 4 phr of quercetin and 15 phr of silica.

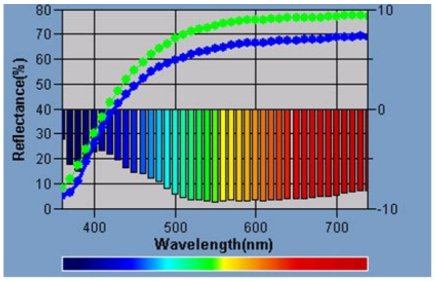

(a) ENR before (green curve) and after solar aging (blue curve)

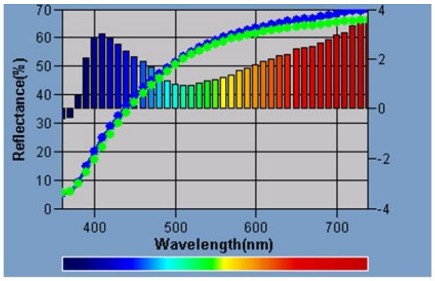

(b) ENR/SIL before (green curve) and after solar aging (blue curve)

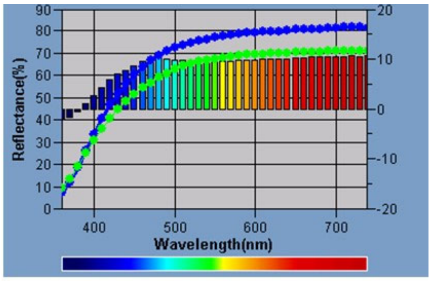

(c) ENR/DCP before (green curve) and after solar aging (blue curve)

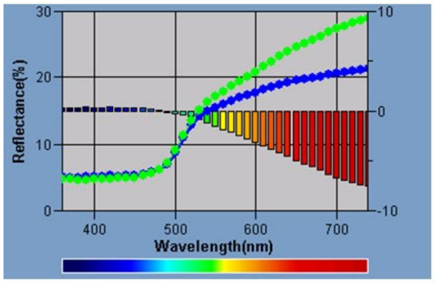

(d) ENR/Q2 before (green curve) and after solar aging (blue curve)

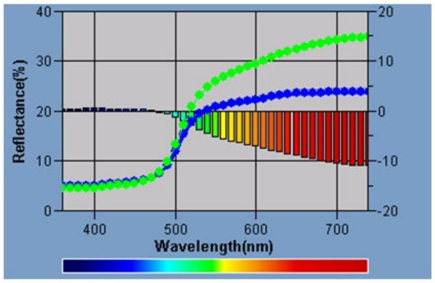

(e) ENR/Q4 before (green curve) and after solar aging (blue curve)

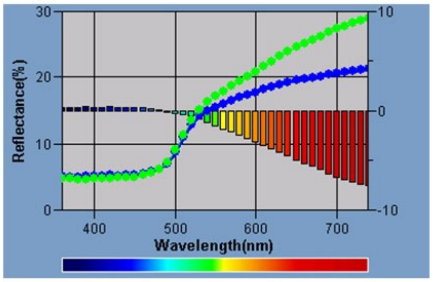

(f) ENR/SIL/Q2 before (green curve) and after solar aging (blue curve)

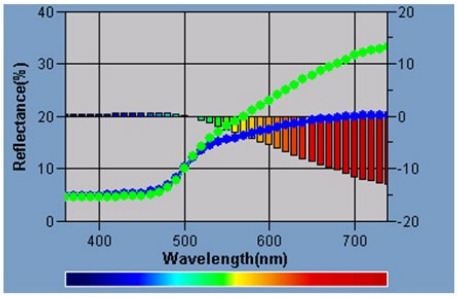

(g) ENR/SIL/Q4 before (green curve) and after solar aging (blue curve)

Figure S3. UV-VIS spectra of ENR-based composites before (green curve) and after solar aging (blue curve): (a) pure uncurred ENR, (b) ENR cured with 15 phr of silica, (c) ENR cured with dicumyl peroxide (DCP), (d) ENR cured with 2 phr of quercetin, (e) ENR cured with 4 phr of quercetin, (f) ENR cured with 2 phr of quercetin and 15 phr of silica, (g) ENR cured with 4 phr of quercetin and 15 phr of silica.
